# 1377. Impact of Pre-Transplant Infectious Diseases Wellness Visit on Vaccine Uptake in Solid Organ Transplant Candidates

**DOI:** 10.1093/ofid/ofab466.1569

**Published:** 2021-12-04

**Authors:** Tommy J Parraga, Jennifer McCorquodale, Sage Greenlee, Zachary Osborn, Zachary W Hanna, Jonathan D Williams, Odaliz Abreu Lanfranco, Ramesh Mayur, George J Alangaden

**Affiliations:** 1 Henry Ford Hospital, Dearborn, Michigan; 2 Michigan State University College of Osteopathic Medicine, Detroit, Michigan; 3 Michigan State University, Berkley, MI; 4 Henry Ford Health System, Detroit, Michigan; 5 Henry Ford Health System, Wayne State University School of Medicine, Detroit, MI

## Abstract

**Background:**

Despite published guidelines, vaccine uptake in solid organ transplant candidates (SOTc) remains suboptimal. We established an Infectious Disease pre-transplant clinic (IDPT) to perform a wellness visit for all SOTc. The visit was conducted by a nurse practitioner (NP) primarily and included pre-transplant assessment and optimization of vaccinations. We report the preliminary results of this pilot project on vaccine uptake in SOTc.

**Methods:**

Retrospective review was done on all SOTc referred to the IDPT from January 2020 to February 2021 at Henry Ford Transplant Institute in Detroit, MI. SOTc were patients listed for different types of transplants. Sociodemographic data, comorbidities, vaccination status for influenza, pneumococcus, hepatitis B, Tdap, Td, and varicella zoster were assessed from electronic medical records and the Michigan Care Improvement Registry that includes vaccination records. Follow up was at least 3 months after IDPT visit. Binomial analysis was performed comparing vaccine uptake in a previous institutional cohort of 530 SOTc from January 2015 to December 2016 in which there was no IDPT visit. Data was analyzed using EpiInfo ver. 7.2.4.0.

**Results:**

A total of 183 SOTc were evaluated in IDPT. Baseline characteristics are shown in Table 1. Median age was 57 years, mean Charlson Comorbidity Index was 4.1. Majority of IDPT visits were done by the NP and most were video visits given the COVID-19 pandemic. Vaccine uptake improved post IDPT for all vaccines and most notably for hepatitis B, varicella zoster and pneumococcal 13V vaccines (Table 2). Of the SOTc, 38 (20.8%) received their vaccines during the IDPT visit or shortly after. Compared to the prior cohort, all vaccines rates improved with the post IDPT visit (p< 0.001) (Table 3).

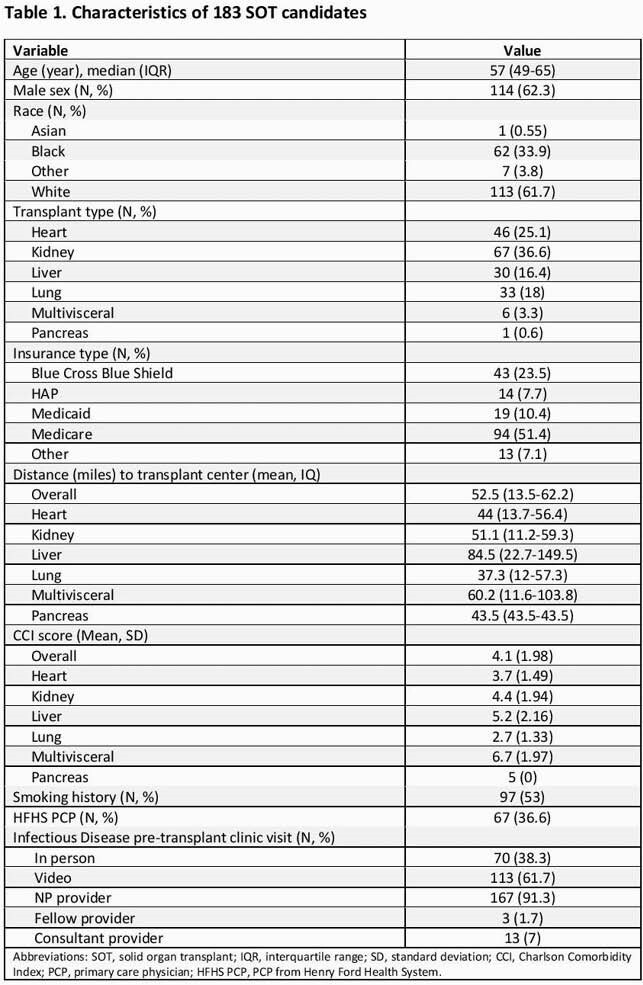

IDPT and vaccines - ID week 2021 - Table 1

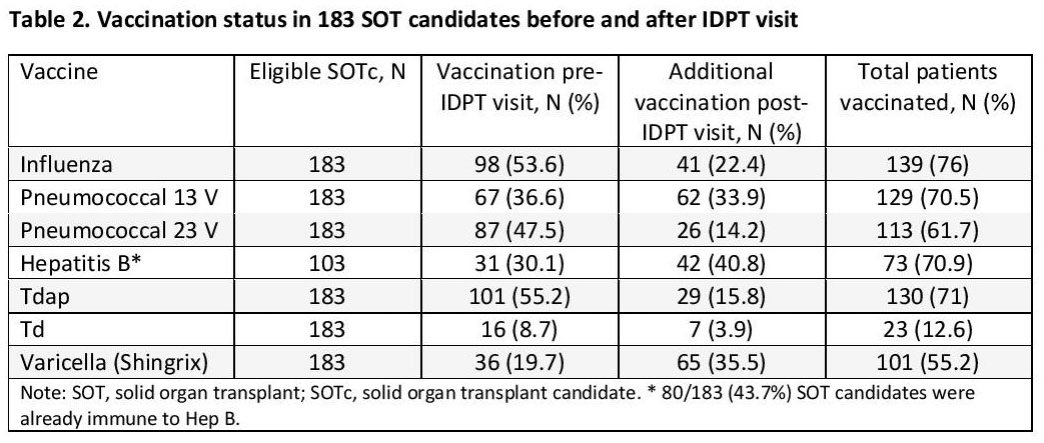

IDPT and vaccines - ID week 2021 - Table 2

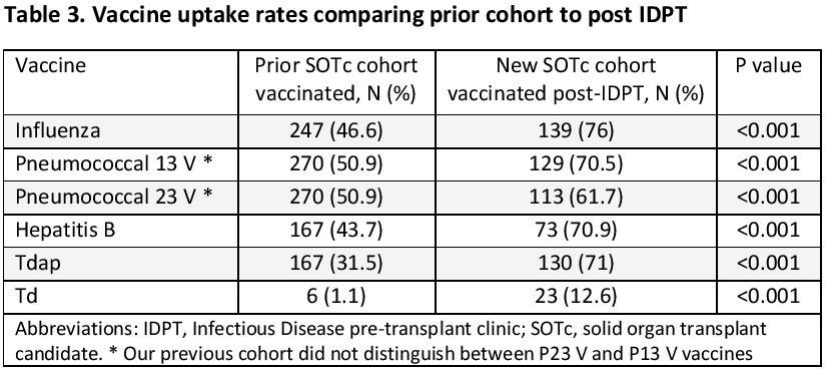

IDPT and vaccines - ID week 2021 - Table 3

**Conclusion:**

IDPT clinic visits significantly improved vaccine uptake in SOTc at our institution. Approximately one in five SOTc had vaccines administered at the time of IDPT visit or shortly after. Implementation of an Infectious Diseases wellness visit as a requirement for all SOTc can provide opportunities to greatly optimize vaccine completion before transplantation.

**Disclosures:**

**All Authors**: No reported disclosures

